# Chemopreventive Potential of *Phyllanthus emblica* Fruit Extract against Colon and Liver Cancer Using a Dual-Organ Rat Carcinogenesis Model

**DOI:** 10.3390/ph17070818

**Published:** 2024-06-21

**Authors:** Chonikarn Singai, Pornsiri Pitchakarn, Sirinya Taya, Warunyoo Phannasorn, Rawiwan Wongpoomchai, Ariyaphong Wongnoppavich

**Affiliations:** 1 Department of Biochemistry, Faculty of Medicine, Chiang Mai University, Chiang Mai 50200, Thailand; chonikarn.s@gmail.com (C.S.); pornsiri.p@cmu.ac.th (P.P.); p.warunyoo@gmail.com (W.P.); rawiwan.wong@cmu.ac.th (R.W.); 2 Functional Food Research Unit, Multidisciplinary Research Institute, Chiang Mai University, Chiang Mai 50200, Thailand; sirinya.t@cmu.ac.th

**Keywords:** anti-mutagenicity, cancer chemoprevention, Indian gooseberry, multi-organ carcinogenesis, multiple primary cancers

## Abstract

Humans are frequently exposed to various carcinogens capable of inducing cancer in multiple organs. *Phyllanthus emblica* (*P. emblica*) is known for its strong antioxidant properties and potential in cancer prevention. However, its effectiveness against combined carcinogens remains relatively unexplored. This study aimed to assess the chemopreventive potential of the ethanolic extract of *P. emblica* fruits against preneoplastic lesions in the liver and colon using a rat model. Rats were administered with diethylnitrosamine (DEN) and 1,2-dimethylhydrazine (DMH) to induce hepato- and colon carcinogenesis, respectively. The ethanolic extract of *P. emblica* fruit at 100 and 500 mg/kg bw significantly reduced the number of preneoplastic lesions in the liver by 74.7% and 55.6%, respectively, and in the colon by 39.2% and 40.8%, respectively. Similarly, the extract decreased the size of preneoplastic lesions in the liver by 75.2% (100 mg/kg bw) and 70.6% (500 mg/kg bw). Furthermore, the extract significantly reduced the cell proliferation marker in the liver by 70.3% (100 mg/kg bw) and 61.54% (500 mg/kg bw), and in the colon by 62.7% (100 mg/kg bw) and 60.5% (500 mg/kg bw). The ethanolic extract also enhanced liver antioxidant enzyme activities and demonstrated free radical scavenging in DPPH, ABTS, and FRAP assays. Additionally, the dichloromethane fraction of *P. emblica* showed significant cancer prevention potential by reducing intracellular ROS and NO production by 61.7% and 35.4%, respectively, in RAW 264.7 macrophages. It also exhibited antimutagenic effects with a reduction of 54.0% against aflatoxin B1 and 52.3% against 2-amino-3,4-dimethylimidazo[4,5-f]quinoline-induced mutagenesis in *Salmonella typhimurium*. Finally, this study highlights the chemopreventive activity of *P. emblica* fruit extract against the initiation of early-stage carcinogenic lesions in the liver and colon in rats treated with dual carcinogens.

## 1. Introduction

Cancer remains a significant health challenge in the 21st century, with a global mortality rate of 48.8% among newly diagnosed cases. Colorectal and liver cancers rank among the leading causes of cancer-related deaths worldwide, accounting for over 900,000 and 700,000 deaths annually, respectively [1]. Notably, more than 90% of cancer incidences are associated with environmental factors, including exposure to carcinogens through various means such as occupation, diet, and lifestyle [2]. For instance, nitrosamines, present in preserved foods and tobacco, have been associated with cancers affecting multiple organs including the liver and lungs [3,4]. Aflatoxin B_1_ (AFB_1_), a common contaminant in various crops, serves as a significant carcinogen linked to liver, lung, and colon cancers [5,6]. Heterocyclic amines found in cooked meats are recognized for increasing the risk of colorectal and urinary tract cancers [7,8]. Additionally, dimethylhydrazine, utilized in military fuel, is known as a carcinogen specifically affecting the colon [9,10].

Normally, individuals are exposed to numerous carcinogenic substances in their daily surroundings, heightening the likelihood of developing cancer across various organs [11,12,13]. This exposure is reflected in the rising prevalence of patients diagnosed with multiple primary cancers, which varies from 2% to 17% [14]. Specifically, colorectal and liver cancers are the most commonly diagnosed multiple primary cancers, with rates of 11.4% and 14.8%, respectively. Individuals with liver cancer encounter an elevated risk of secondary primary cancers in the liver, as well as in the colorectal and lung regions. Similarly, colorectal cancer shows a strong correlation with subsequent diagnoses of colorectal, liver, and lung cancers [15].

To address these challenges, researchers have developed multi-organ carcinogenesis models over the years, aiming to replicate real-world exposure to carcinogens and investigate the impact of environmental chemicals on cancer development across various organs within a single experimental framework. These models operate on the initiation–promotion concept of chemical carcinogenesis, employing multiple initiator carcinogens to evaluate the potential of environmental chemicals in promoting cancer development across multiple organs [16,17,18,19]. For instance, studies have demonstrated that the combined administration of *N*-nitroso diethylamine (NDEA) and azoxymethane (AOM) to initiate both liver and colon carcinogenesis results in more severe preneoplastic lesions in the liver compared to NDEA treatment alone [20]. Similarly, the combined action of diethylnitrosamine (DEN) and 1,2-dimethylhydrazine (DMH) in rat models promotes hepatocarcinogenesis by inducing metabolic enzymes and enhancing DNA mutation rates [21].

Over the years, multi-organ carcinogenesis models have received significant attention for assessing chemopreventive agents, which are either natural or synthetic substances that intervene at different stages of the multistep carcinogenesis process [22]. Interestingly, the effectiveness of natural products in these multi-organ models often deviates from their action in single-carcinogen treatments. For instance, green tea catechins have been shown to reduce colon carcinogenesis induced by DMH in rats and liver tumors in mice caused by DEN [23]. However, in multi-carcinogen models treated with a combination of DEN, DMH, MNU, BBN, and DHPN, these catechins only exhibit a reduction in small intestine tumors, failing to show beneficial effects on lung and colon tumors, and slightly enhancing hepatocarcinogenesis [24]. Similarly, bovine lactoferrin, a milk component effective against preneoplastic lesions in DEN-induced liver carcinogenesis in rats, has no influence on cancer development in the liver, kidney, and thyroid when induced by DEN, DHPN, and MNBA in multi-organ models [25,26]. Thus, comprehensively understanding the activity of natural products in complex models presents significant challenges in interpreting their efficacy across various cancer scenarios.

*Phyllanthus emblica* Linn., known as *Indian gooseberry* or amla, stands out as a promising natural product in cancer prevention. Indigenous to tropical southeast Asia, this plant has long been used in traditional medicine for treating a variety of illnesses such as peptic ulcers, diabetes, helminthic infections, and inflammation [27]. Renowned for its nutritional richness, particularly high levels of Vitamin C, and array of bioactive compounds such as phenolic acids and tannins, the fruit of *P. emblica* offers great health benefits [28]. In addition to its nutritional value, this fruits possesses a wide range of pharmacological properties including antioxidant, anti-inflammatory, immunomodulatory, hepatoprotective, and anticancer activities [29,30]. Extracts from *P. emblica* have previously demonstrated promising effects against hepatocarcinogenesis models, both in vitro and in vivo [31,32,33,34,35]. It appears to intervene at the initial stages of carcinogenesis by enhancing the activity of liver antioxidant enzymes, such as glutathione (GSH), glutathione peroxidase (GPx), glutathione reductase (GR), and the detoxifying enzyme glutathione-*S*-transferase (GST), in mice exposed to the DMBA carcinogen. It also reduces the levels of hepatic activating enzymes such as cytochrome P450, thereby decreasing the formation of carcinogen-induced DNA adducts and gene mutation in models induced by AFB_1_ and benzo[a]pyrene [36,37]. Furthermore, *P. emblica* extract has demonstrated the ability to prevent the progression of initiated cells to more advanced stages by inducing apoptosis in mice induced by DEN and human liver cancer cell lines [34]. In addition to its effects on liver cancer, the extract also inhibits the initiation of colon cancer by enhancing DNA repair mechanisms in human colon carcinoma cell lines, including HT29 and HCT116 [38]. It further impedes the promotion and progression phase of colon cancer by reducing cell proliferation and inducing apoptosis in various types of colon cancer cell lines [31,39,40,41].

Although the chemopreventive properties of *P. emblica* fruits are well-documented in various liver and colon carcinogenesis models, their effectiveness in more complex models with co-carcinogen exposures has yet to be explored. Thus, this study aims to investigate the influence of *P. emblica* fruits on a dual-carcinogenesis model, combining well-known carcinogens such as DEN-induced liver and DMH-induced colon carcinogenesis. Furthermore, the study seeks to assess phytochemical contents and the antioxidant, anti-inflammatory, and antimutagenic effects of the ethanolic extract and its solvent fractions to elucidate potential mechanisms and bioactive compounds that may contribute to the prevention of the early stage of carcinogenesis.

## 2. Results

### 2.1. Crude Ethanolic Extract of P. emblica Fruit Reduces the Formation of Preneoplastic Lesions in the Liver and Colon of Rats Treated with Dual Carcinogens

**Figure 1:**
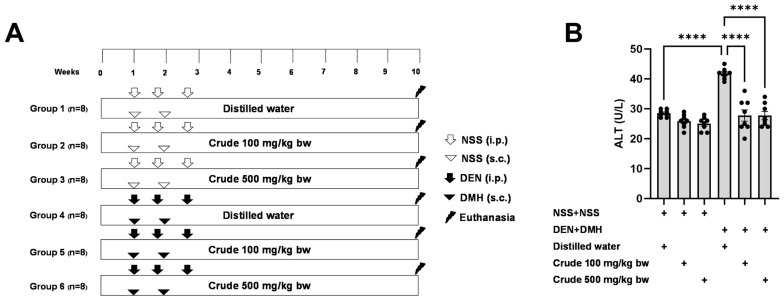
(**A**) Overview of the study design and the timeline for the administration of the carcinogens (DEN and DMH) and crude *P. emblica* fruit extract in rats. (**B**) Serum alanine aminotransferase (ALT) levels in rats. Data are expressed as mean ± standard deviation (n = 8). **** *p*  ≤  0.0001 indicates a statistical significance.

**Figure 2:**
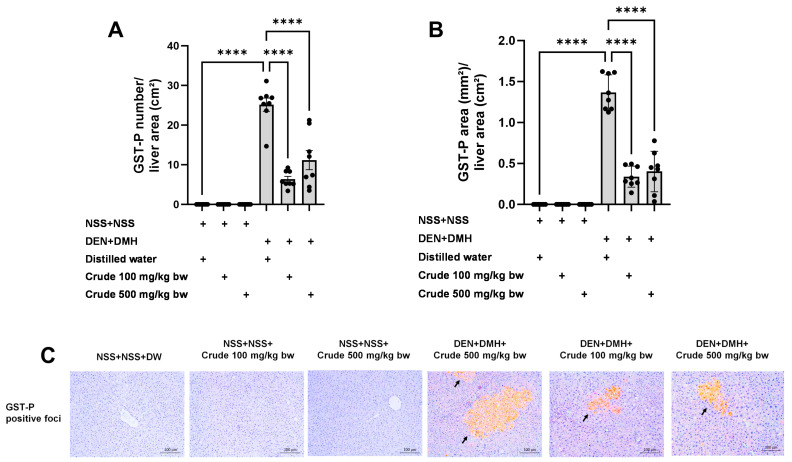
Effects of 10-week administration of crude *P. emblica* fruit extract on dual carcinogen-induced pre-neoplastic lesions in rat liver. Analysis of preneoplastic lesions includes (**A**) the number and (**B**) the area of liver glutathione *S*-transferase placental form (GST-P) positive foci, as detected by immunohistochemistry. (**C**) Representative images of preneoplastic lesions (GST-P positive foci as arrow indicated) in the liver (200× magnification). Data are expressed as mean ± standard deviation (n = 8). **** *p*  ≤  0.0001 indicates a statistical significance.

**Figure 3:**
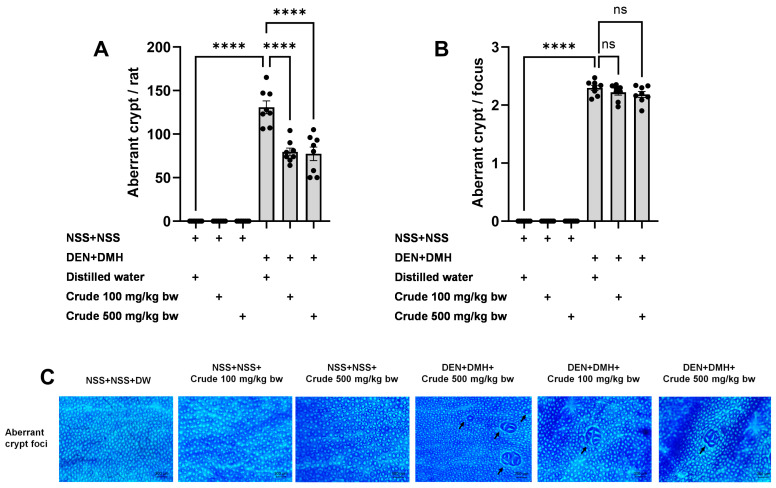
Effects of 10-week administration of crude *P. emblica* fruit extract on dual carcinogen-induced pre-neoplastic lesions in rat colon. Analysis of preneoplastic lesions includes (**A**) the number and (**B**) the size of aberrant crypts in the colon, identified by 0.2% methylene blue staining. (**C**) Representative images of preneoplastic lesions (Aberrant crypt foci as indicated by arrows) in the colon (100× magnification). Data are expressed as mean ± standard deviation (n = 8). **** *p*  ≤  0.0001 indicates a statistical significance, “ns” indicates not significant (*p* > 0.05).

The effectiveness of the crude extract in reducing preneoplastic lesions in the liver and colon was investigated in rats exposed to two carcinogens simultaneously (Figure 1A). After injection of DEN and DMH, rats had significantly increased serum ALT levels and preneoplastic lesions, including GST-P positive foci in the liver and aberrant crypt foci in the colon, compared to the group receiving NSS alone (Figure 1, Figure 2 and Figure 3). The administration of the crude extract at both 100 and 500 mg/kg bw significantly lowered serum ALT levels (33.53%) in rats treated with carcinogens (Figure 1B), indicating the protection ability of the extract against carcinogen-induced hepatic damage. Moreover, treatment with the extract at both 100 and 500 mg/kg bw markedly reduced the number of hepatic GST-P positive foci by 74.7% and 55.6%, respectively, and colonic aberrant crypts by 39.2% and 40.8%, respectively, in rats treated with carcinogens. (Figure 2A and Figure 3A), suggesting its effective prevention of preneoplastic lesions initiation in the liver and colon following carcinogen exposure. Furthermore, in rats treated with carcinogens, administering crude extract at both 100 and 500 mg/kg bw significantly reduced the area of GST-P positive foci in the liver by 75.2% and 70.6%, respectively, while the size of colonic aberrant crypt foci remained unchanged (Figure 2B and Figure 3B). These findings indicate the effectiveness of the crude extract in controlling lesion growth in the liver but not in preventing the progression of lesions in the colon during the early stages of carcinogenesis. Importantly, administration of the crude extract at the tested doses did not elevate ALT levels, nor induce hepatic GST-P positive foci or colonic aberrant crypt foci, suggesting its non-carcinogenicity in rat liver and colon (Figure 1, Figure 2 and Figure 3).

### 2.2. The Crude Ethanolic Extract of P. emblica Fruit Decreases Cell Proliferation during the Early Stages of Liver and Colon Carcinogenesis in Rats

**Figure 4:**
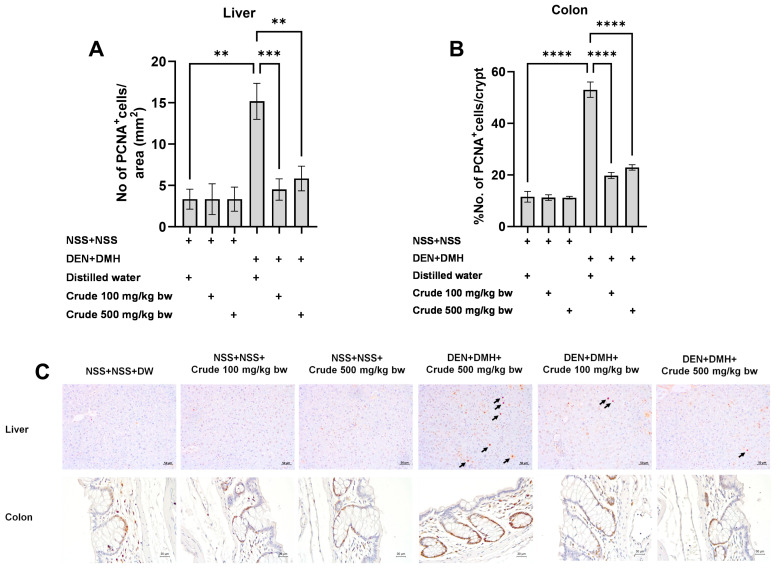
Effect of crude *P. emblica* fruit extract on the early-stage carcinogenesis in rats: modulation of cell proliferation. Immunohistochemical staining quantified (**A**) PCNA-positive cells in the liver, (**B**) PCNA-positive cells in the colon. (**C**) Representative immunohistochemical images of PCNA-positive cells in the liver sections (200× magnification) as indicated by arrows and the colon sections (400× magnification). PCNA-positive hepatic cells are indicated by arrows, while the PCNA-positive cells in the colon exhibit brownish staining. Data are expressed as mean ± standard deviation. Levels of statistical significance are denoted as ** *p* ≤ 0.01, *** *p* ≤ 0.001, **** *p* ≤ 0.0001.

**Figure 5:**
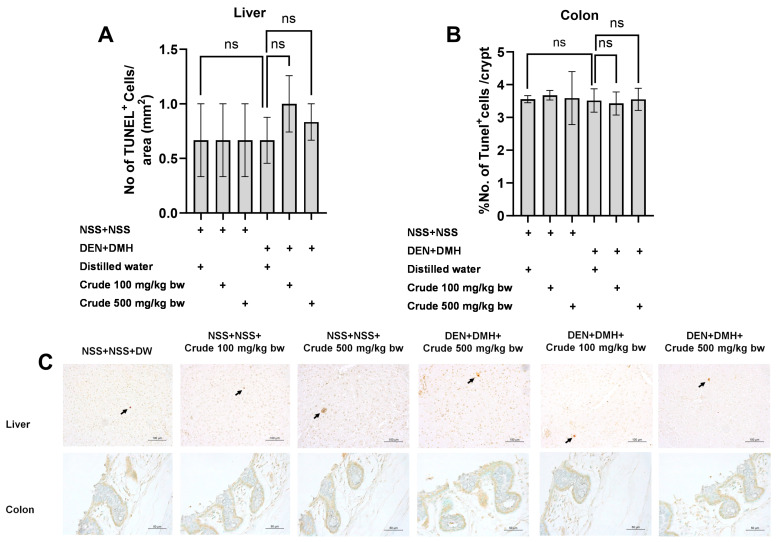
Effect of crude *P. emblica* fruit extract on the early-stage carcinogenesis in rats: modulation of cell apoptosis. Immunohistochemical staining quantified (**A**) TUNEL-positive cells in the liver and (**B**) TUNEL-positive cells in the colon. (**C**) Representative immunohistochemical images of TUNEL-positive cells in the liver (200× magnification) and the colon (400× magnification) sections. TUNEL-positive hepatic cells are indicated by arrows, while the TUNEL-positive cells in the colon exhibit brownish staining. Data are expressed as mean ± standard deviation. “ns” indicates not significant (*p* > 0.05).

Based on the effects of the crude extract on preneoplastic lesions in the liver and colon of rats treated with carcinogens, the underlying mechanisms involving cellular proliferation and apoptosis were further investigated. Treatment with the crude extract alone showed no significant impact on cell proliferation or apoptosis in normal rats, in both liver and colon tissues (Figure 4 and Figure 5). Rats treated with DEN and DMH exhibited a significant increase in the number of PCNA^+^ cells, indicating cell proliferation, in both the liver and colon compared to the NSS-treated group. Administration of the crude extract at doses of 100 and 500 mg/kg bw significantly reduced the number of PCNA^+^ cells in both the liver by 70.3% (100 mg/kg) and 61.54% (500 mg/kg), and the colon by 62.7% (100 mg/kg) and 60.5% (500 mg/kg) of carcinogen-treated rats (Figure 4). However, the treatment did not result in any changes in the number of TUNEL^+^ cells, which is an indicator of apoptotic cells, in either the liver or colon of carcinogen-treated rats (Figure 5). These findings suggest that crude extraction of *P. emblica* fruit primarily inhibits the formation and progression of preneoplastic lesions in the early stages of carcinogenesis by modulating cell proliferation rather than affecting apoptosis.

### 2.3. The Crude Ethanolic Extract of P. emblica Fruit Enhances the Key Antioxidant Enzymes and Non-Enzymatic Molecules in Rat Liver

**Figure 6:**
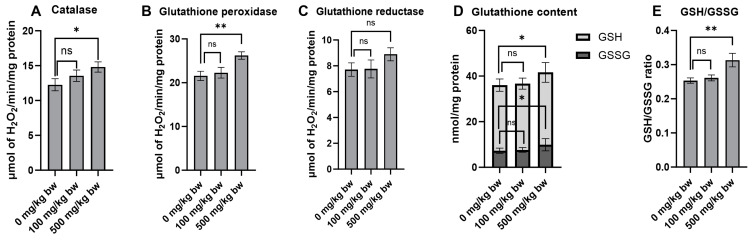
The antioxidant enzyme activities and glutathione levels in the liver cytosolic fraction of normal rats following treatment with crude ethanolic extract of *P. emblica* fruit. The activities of antioxidant enzymes include (**A**) catalase, (**B**) glutathione peroxidase, and (**C**) glutathione reductase. The quantification of non-enzymatic molecules includes (**D**) total glutathione levels and (**E**) the ratio of reduced (GSH) to oxidized (GSSG) glutathione in the liver cytosolic fraction. Measurements were performed using specific assays for each enzyme and molecule. Data are presented as mean ± standard deviation. Statistical significance is denoted as * *p* ≤ 0.05 and ** *p* ≤ 0.01 in comparison to the vehicle control group. “ns” indicates not significant (*p* > 0.05).

Antioxidants play a crucial role in cancer prevention by counteracting oxidative stress and cellular damage, which are prominent risk factors for the formation of preneoplastic lesions in the early stages of carcinogenesis. The effect of the crude extract on key antioxidant enzymes and non-enzymatic molecules was evaluated in normal rat livers (Groups 4–6). Treatment of the rats with 500 mg/kg bw of the crude extract for ten weeks markedly increased the activities of catalase and glutathione peroxidase compared to the vehicle control group (Figure 6A,B). While there was no significant change in glutathione reductase activity, there was even a trend toward increased activity in rats treated with a high dose of the crude extract (Figure 6C). Furthermore, treatment of the rats with a high dose of the crude extract significantly increased the levels of non-enzymatic molecules, including reduced glutathione (GSH), oxidized glutathione (GSSG), and the GSH/GSSG ratio (Figure 6D,E). These findings highlight the considerable potential of the crude extract in indirectly boosting antioxidant defenses by stimulating the activity of antioxidant enzymes and increasing the levels of non-enzymatic molecules.

### 2.4. Phytochemical Analysis of Crude Ethanolic Extract from P. emblica Fruit

To investigate potential compounds within the crude extract that could prevent the early stages of carcinogenesis, a phytochemical analysis was conducted via sequential solvent partitioning. The obtained yields were 0.98 ± 0.1% for HEX, 0.44 ± 0.1% for DCM, 13.02 ± 4.4% for EAC, 20.07 ± 1.8% for BA, and 20.99 ± 0.1% for the residue fraction. Phytochemical screening of the crude extract revealed a significant presence of polyphenols, tannins, and flavonoids (Table S1). Further spectrophotometric assessments revealed a significantly high concentration of condensed tannins in the crude extract, whereas the EAC fraction exhibited elevated levels of phenolic compounds, flavonoids, and hydrolysable tannins (Table 1). Additionally, the phytochemical profile of each fraction was obtained from HPLC analysis. Catechin and epicatechin were predominantly found in the crude extract, while the DCM fraction contained abundant *trans*-cinnamic acid and ellagic acid. The EAC fraction exhibited high levels of gallic acid and syringic acid, while the BA fraction showed a substantial content of rutin (Table 1, Figures S1–S3).

### 2.5. The Crude Ethanolic Extract of P. emblica Fruit and Its Fractions Exhibit Free Radical Scavenging Activities

The crude extract and its fractions were assessed for their ability to neutralize free radicals generated from the metabolism of carcinogens, which play a crucial role in genetic mutation and cancer promotion. DPPH, ABTS, and FRAP assays were conducted to evaluate their free radical scavenging capabilities through different mechanisms. The results indicated that both the crude extract and its fractions exhibited potent free radical scavenging activities. Notably, the EAC fraction displayed remarkable efficacy, exhibiting the lowest IC_50_ values in both DPPH and ABTS assays, indicating its ability to neutralize free radicals by donating a hydrogen atom and electron, respectively. Moreover, EAC showed the highest effectiveness in the FRAP assay, indicating its strong reducing ability (Table 2). These findings highlight the antioxidant potential of the *P. emblica* crude extract, especially its EAC fraction, in neutralizing free radicals.

### 2.6. DCM Fraction from the Crude Ethanolic Extract Attenuates Intracellular ROS and NO Production in RAW 264.7 Macrophage Cell Lines

**Figure 7:**
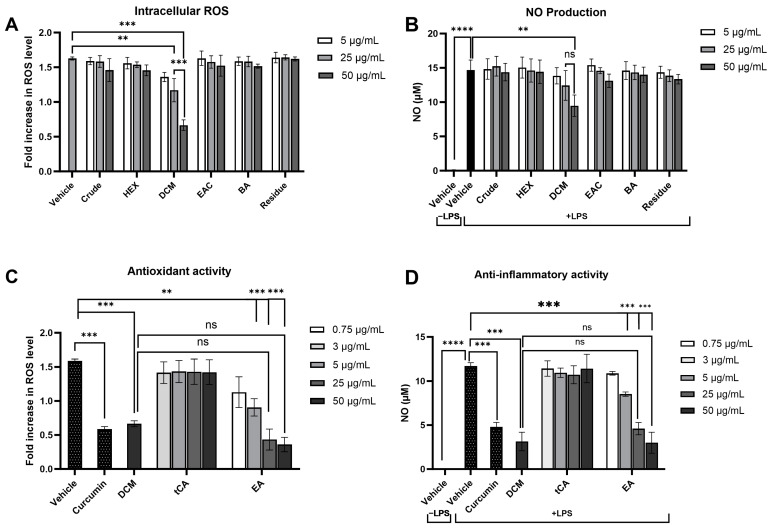
Effects of *P. emblica* crude ethanolic extract fractions and their potential leading bioactive compounds on oxidative stress and inflammatory responses in lipopolysaccharide-stimulated RAW 264.7 macrophage cell lines. The impact of *P. emblica* crude ethanolic extract and its fractions on (**A**) intracellular reactive oxygen species (ROS) production, as measured by DCFH-DA staining, and (**B**) nitric oxide (NO) production, assessed using Griess reagents. The effects of their potential leading bioactive compounds on (**C**) intracellular ROS production and (**D**) NO production. Curcumin at 2 μg/mL served as a positive control. Results are presented as mean ± standard deviation, based on data from three independent experiments. Significance levels are marked as ** *p* ≤ 0.01, *** *p* ≤ 0.001, **** *p* ≤ 0.0001. “ns” indicates not significant (*p* > 0.05). tCA: *trans*-cinnamic acid, EA: Ellagic acid.

Modulating the response of macrophages to reduce oxidative stress and inflammation is critical in preventing damage to cellular components and the development of cancer. The effect of the crude extract and its fractions on intracellular ROS and NO production, key pro-inflammatory mediators, was evaluated in LPS-induced RAW 264.7 macrophages. Preliminary cytotoxicity tests using the MTT assay showed that concentrations up to 100 μg/mL of each fraction did not affect cell viability (Table S2). Following LPS treatment, intracellular ROS and NO levels were significantly increased. Notably, only treatment with the DCM fraction at concentrations of 25 and 50 μg/mL significantly attenuated LPS-induced ROS levels by 28.1% and 61.7%, respectively, in a dose-dependent manner compared to the vehicle control (Figure 7A), highlighting the pivotal roles of the substances in the DCM fraction in preventing intracellular oxidative stress. Similarly, the DCM fraction at 50 µg/mL markedly lowered nitrite levels (35.4%), indicative of reduced NO production, while other fractions did not demonstrate a significant change compared to cells treated with LPS alone (Figure 7B). These results suggest that the DCM fraction from the crude extract can effectively attenuate the inflammatory response by diminishing ROS and NO levels produced by inflammatory cells.

Based on the effectiveness of the DCM fraction in reducing intracellular ROS and NO production, further analysis was conducted to identify the potential key bioactive compounds within this fraction. *Trans*-cinnamic acid and ellagic acid, which are predominantly found in the DCM fraction, were selected for further investigation. The antioxidant and anti-inflammatory capabilities of these compounds were evaluated at concentrations corresponding to their levels found in the DCM fraction as determined by HPLC analysis. Treatment with 3 μg/mL of *trans*-cinnamic acid or 0.75 μg/mL of ellagic acid showed lower antioxidant and anti-inflammatory efficacies than the DCM fraction (50 μg/mL) (Figure 7C,7D). These findings suggest that the reduction in ROS and NO production is not only attributed to one compound but rather to the interaction among multiple compounds within the DCM fraction of the crude ethanolic extract of *P. emblica.*

### 2.7. The Crude Ethanolic Extract of P. emblica Fruit and Its Fractions Demonstrate Anti-Mutagenic Activity in Salmonella typhimurium 

Gene mutations induced by carcinogens are a fundamental step in cancer initiation, leading to abnormal cell growth and proliferation. The anti-mutagenic effect of the crude extract and its fractions were evaluated on *S. typhimurium* strains TA98 and TA100, which are sensitive to frameshift mutations and base-pair substitutions, respectively. These evaluations were carried out with and without metabolic activation (S9 mix). The results showed that the number of revertant colonies produced by the crude extract and its fractions at concentrations up to 5 mg/plate did not significantly differ from those produced by the vehicle control. Moreover, the highest concentrations of each fraction did not exhibit cytotoxicity to bacterial growth (Table 3). These data indicate that all fractions are non-mutagenic and non-toxic toward both strains, regardless of metabolic activation. The potential antimutagenic effects of each fraction against standard mutagens were further explored with non-toxic doses. In the presence of metabolic activation, the crude extract and its fractions at 0.2 mg/plate significantly reduced the number of revertant colonies in both bacterial strains compared to the mutagen control when exposed to indirect mutagens (AFB_1_ and MeIQ), indicating their ability to inhibit gene mutation induced by indirect mutagens. Notably, the DCM fraction at 0.04 mg/plate exhibited 54.0% inhibition in TA98 and 52.3% in TA100, marking the highest level of antimutagenicity among other fractions. This suggests that the crude extract, particularly the DCM fraction, prevents frameshift mutations and point mutations induced by indirect mutagens through the modulation of xenobiotic metabolizing enzymes. However, in the absence of metabolic activation, none of the fractions displayed antimutagenic activity against direct-acting mutagens, including AF-2 and NaN_3_, in both bacterial strains, suggesting specificity in their action mechanism which may involve the biotransformation (Table 4).

Similarly, the results suggest that the DCM fraction is the most promising in preventing gene mutations in bacterial mutation tests. Consequently, *trans*-cinnamic acid and ellagic acid, present in high concentrations in the DCM fraction, were selected for further investigation. To determine their anti-mutagenic potential, the doses of *trans*-cinnamic acid and ellagic acid were calculated based on their concentrations in the DCM fraction as detected by HPLC. In the *Salmonella* mutation assay, 0.01 mg/plate of *trans*-cinnamic acid and 0.003 mg/plate of ellagic acid exhibited weaker inhibitory potential against AFB_1_ and MeIQ compared to 0.2 mg/plate of the DCM fraction. These findings suggest that the ability to prevent gene mutations of DCM fraction derived from crude ethanolic extract of *P. emblica* might be due to the interactions among various constituents (Table 5).

## 3. Discussion

The utilization of a dual-carcinogens model underscores the complex nature of human carcinogenesis. *Phyllanthus emblica* (*P. emblica*) fruits have demonstrated the premise of inhibiting or delaying the multistep process of carcinogen-induced carcinogenesis across various cancer types [42]. Nonetheless, the effectiveness of *P. emblica* fruit extracts against multiple carcinogens has remained unexplored. The present study unveils the chemopreventive potential of *P. emblica* fruit extracts in a model using dual carcinogens. Significantly, this model demonstrates for the first time the capability of the crude ethanolic extract of *P. emblica* fruit against the preneoplastic lesions in both the liver and colon of rats during the early stage of carcinogenesis. This effect is primarily mediated by modulating key cellular processes such as proliferation, while apoptosis is not markedly impacted. Moreover, the study confirms the potential mechanisms of this plant in preventing carcinogenesis, including enhanced activities of antioxidant enzymes, robust scavenging of free radicals, reduced inflammation, and inhibition of carcinogen-induced gene mutations, particularly through the potential active compounds in the DCM fraction.

The early stages of carcinogenesis involve the development of preneoplastic lesions, which occur between the initiation and promotion phases [43]. In this study, DEN and DMH, considered a complete carcinogen, were employed to induce early-stage hepatic and colonic carcinogenesis. These carcinogens are metabolically activated via CYP450 2E1, generating DNA-reactive metabolites that increase the risk of mutations and preneoplastic lesions in both the liver and colon [44,45]. The findings from the dual-organ model suggest that the ethanolic extract of *P. emblica* fruit holds significant potential in preventing the formation of preneoplastic lesions during the early stages of both hepatic and colonic carcinogenesis, thus demonstrating the strong cancer chemopreventive properties of *P. emblica* fruit against this model. However, its effects are more pronounced in the liver, as evidenced by reductions in both the number and size of GST-P positive foci, whereas in the colon, only a reduction in the number of ACF is observed. This underscores the role of *P. emblica* fruit in regulating the initiation and growth of preneoplastic lesions in the liver following exposure to co-carcinogens in rats, while only preventing the initiation of lesions in the colon. This distinct impact is attributed to the ability of *P. emblica* fruit to enhance key antioxidant enzymes and non-enzymatic molecules in the rat liver, a crucial organ containing various enzymes involved in carcinogen-induced carcinogenesis. These activities help counteract DNA-reactive metabolites from DEN-induced preneoplastic lesions in the liver, leading to a more pronounced effect in the liver compared to the colon. Typically, these antioxidants decrease during cancer development [46], emphasizing the potential of *P. emblica* fruit as an indirect antioxidative agent, as previously reported [36]. Moreover, *P. emblica* fruit, especially its EAC fraction, shows a direct effect of scavenging free radicals in vitro. This aligns with previous research showing a positive connection between plant phenolic compounds and antioxidants [47]. EAC is rich in phenolic compounds compared to the other fractions, especially gallic acid and syringic acid, known for their direct antioxidant properties due to multiple phenolic hydroxyl and methoxy groups [48,49]. These phenolic compounds in *P. emblica* fruit extract are most likely effective at directly scavenging free radicals.

*P. emblica* fruit extract, particularly the DCM fractions, is effective in preventing genetic mutations induced by carcinogens. This prevention occurs not through the direct neutralization of mutagens or impacting the DNA repair system but by modulating xenobiotic-metabolizing enzymes such as CYP450 in liver fractions that can metabolize carcinogen to active DNA-reactive metabolites. This agrees with previous findings where *P. emblica* extracts significantly reduced the mutagenicity of known indirect carcinogens such as aflatoxin B_1_ and benzo[a]pyrene in the Ames test [37]. Additionally, the study highlights that DCM mainly contains *trans*-cinnamic acid (tCA) and ellagic acid (EA). t-CA has been reported to possess potential as an antimutagenic agent against indirect mutagens [50]. Moreover, EA also exerts its anti-mutagenic effects by modulating the activity of CYP450 enzymes, leading to decreased activation of numerous xenobiotics into toxic metabolites, ultimately resulting in a reduction in gene mutations [51]. However, when EA and tCA are studied individually, their ability to prevent mutations is weaker compared to the DCM extract. Importantly, lower concentrations of EA and tCA were employed in this study compared to previous research [52,53]. Interestingly, the activities of EA and tCA improved with increasing concentrations, suggesting a dose-dependent relationship. Notably, DCM is not only composed of EA and tCA; it also contains a variety of other beneficial compounds such as gallic acid, syringic acid, and epicatechin, which contribute to preventing carcinogenesis [54]. This implies that a synergistic effect of various compounds within the DCM fraction of *P. emblica* fruit extract enhances their protective actions as antimutagenic agents against indirect mutagens that require metabolic activation. Taken together, *P. emblica* fruit extracts have the potential to prevent initiated cell and preneoplastic lesion formation in the liver and colon during the early stage of carcinogenesis by modulating the metabolism of carcinogens and increasing the oxidative stress defense mechanism.

An imbalance between cell proliferation and apoptosis is a critical factor in the development and progression of preneoplastic lesions, potentially leading to the accumulation of cells with genetic changes, a significant step toward the development of cancer [55]. This study found a significant impact of *P. emblica* fruit on cell proliferation but did not find a significant effect on cell apoptosis in preneoplastic lesions in both the liver and colon. However, previous reports have indicated the apoptosis-inducing capacity of *P. emblica* [34]. The absence of this ability in our study might be due to the robust model of co-carcinogen treatment, inducing more severe effects compared to previous studies involving single carcinogens [21,56,57]. After carcinogen exposure, the initiated cells tend to maintain elevated levels of reactive oxygen species (ROS) and nitric oxide (NO), contributing to an inflammatory response that creates a favorable microenvironment for tumor development. This event further exacerbates DNA damage and promotes uncontrolled cell proliferation, leading to the formation of early preneoplastic lesions [58]. Macrophages play a significant role in the tumor microenvironment of carcinogenesis [59]. Our study showed that *P. emblica* fruit extract, particularly the DCM fraction, regulates macrophage activity by reducing ROS and NO production in LPS-induced macrophages. As prominent compounds in the DCM fraction, EA and tCA have been shown to reduce NO production by diminishing the expression of iNOS protein [53,60]. Moreover, previous studies showed that both compounds enhance intracellular antioxidant enzymes by activating Nrf2-mediated antioxidant genes, resulting in reduced intracellular ROS production [61,62]. However, our research suggests that the potential of EA and tCA alone appears to be weaker compared to DCM. It is noteworthy that combining various compounds has been demonstrated to enhance the activity of pharmaceuticals and mitigate the side effects of single compounds, as substantiated by prior research [63]. Ultimately, the modulation of the tumor environment by *P. emblica* fruit extract, as reported in this study, contributes to the prevention of initiated cell proliferation into preneoplastic lesions in the liver and colon of rats treated with dual carcinogens in early carcinogenesis.

## 4. Materials and Methods

### 4.1. Materials and Chemicals

All chemicals employed in the extraction process were purchased from RCI Labscan Ltd. (Bangkok, Thailand). The RAW 264.7 cell line was sourced from the American Type Culture Collection (ATCC, Manassas, VA, USA). The standard mutagens, including 2-aminoanthracene (2-AA), 2-(2-furyl)-3-(5-nitro-2-furyl) acrylamide (AF-2), and 2-amino-3,4-dimethylimidazo[4,5-f]quinoline (MeIQ), were obtained from Wako Pure Chemicals Industries Ltd. (Osaka, Japan). Lipopolysaccharides (LPS) (*Escherichia coli* O111:B4), 2′,7′-dichlorofluorescein-diacetate (DCFHDA), and other mutagens, including aflatoxins B_1_ (AFB_1_), sodium azide (NaN_3_), 2-aminoflurene (2-AF), Diethylnitrosamine (DEN), and 3,3′-diaminobenzidine (DAB), were purchased from Sigma-Aldrich (St. Louis, MO, USA). 1,2-Dimethylhydrazine (DMH) was sourced from TCI (Tokyo, Japan). The avidin-biotin–horseradish peroxidase complex (ABC) kit and Vectastain Elite ABC kit (Universal) were obtained from Vector Laboratories Inc. (Burlingame, CA, USA). The Apoptag detection kit was purchased from Merck Millipore (Darmstadt, Germany).

### 4.2. Preparation of P. emblica Fruits Extract

*P. emblica* fruits were cultivated and harvested in Chai Badan District, Lopburi, Thailand, and then processed and subjected to quality control procedures following applicable guidelines and regulations managed by Prolac Co., Ltd. (Lamphun, Thailand). The *P. emblica* fruit powder, obtained commercially from Prolac Co., Ltd., underwent extraction twice with 70% ethanol over two days. Subsequently, the crude extract of *P. emblica* was filtrated and dried. This crude extract was then partitioned using various solvents, including hexane, dichloromethane, ethyl acetate, and butanol, in 1:1 ratio, yielding hexane (HEX), dichloromethane (DCM), ethyl acetate (EAC), butanol (BA), and residue fractions, respectively. All fractions were subjected to evaporation and freeze-drying and stored in a −20 °C refrigerator until further analysis.

### 4.3. Animals Model

All Wistar rats were obtained from Nomura Siam International Co, Ltd., Bangkok, Thailand. They were housed in the Animal House, Faculty of Medicine, Chiang Mai University, Chiang Mai, Thailand, under standard environmental conditions of temperature at 25 °C under a 12 h dark/light cycle and were allowed free excess to a pellet diet and tap water throughout the experiments. The oral dose used in these experiments was determined based on the results of the acute oral toxicity test, performed following the procedures outlined in OECD Guideline No. 425 [64]. The animal experimental design was approved by the Animal Ethics Committee of the Faculty of Medicine, Chiang Mai University (Protocol No. 07/2563, approved on 17 April 2020 and No.20/2564, approved on 26 August 2021). All methods were performed following the relevant guidelines and regulations. This study follows the recommendation in the ARRIVE guidelines.

### 4.4. Carcinogenicity and Anti-Carcinogenicity

The carcinogenic and anti-carcinogenic effects of *P. emblica* fruit were investigated using a dual-organ carcinogenicity model following the method described by Punvittayagul et al., 2019 [21]. Four-week-old male rats were randomly divided into six groups, each consisting of eight rats. Groups 1 and 4 were fed by gavage with distilled water five days a week, serving as the vehicle control. Groups 2 and 5 were fed by gavage with 100 mg/kg bw of crude extract five days a week, while Groups 3 and 6 were fed by gavage with 500 mg/kg bw of crude extract. Groups 4 to 6 were intraperitoneally injected (i.p.) with 100 mg/kg bw of DEN three times on days 7, 11, and 18 to induce hepatocarcinogenesis. Additionally, on days 7 and 14, these same groups received subcutaneous injections (s.c.) of DMH to induce colorectal carcinogenesis. In contrast, Groups 1 to 3 received intraperitoneal and subcutaneous injections of 0.9% normal saline (NSS) instead of DEN and DMH. After eleven weeks, the rats were humanely euthanized. Blood samples were collected for serum alanine aminotransferase (ALT) level measurement by the Small Animal Hospital at Chiang Mai University, Thailand. Furthermore, both the liver and colon tissues were collected to assess preneoplastic lesions and investigate underlying mechanisms.

### 4.5. Determination of Preneoplastic Lesions in Liver and Colon

The presence of hepatic glutathione *S*-transferase placental form (GST-P) positive foci was determined using an immunohistochemical staining technique [65]. Liver sections were initially deparaffinized and then rehydrated. Following immersion in a solution containing 3% H_2_O_2_ and skimmed milk, the slides were incubated with rabbit polyclonal anti-rat GST-P antibody (Nagoya, Japan). Subsequently, an ABC kit was employed to develop brown coloration using DAB, followed by counterstaining with hematoxylin. The numbers and sizes of GST-P positive foci were quantified using the Leica Application Suite (LAS) Interactive Measurement program (Leica Microsystems, Wetzlar, Germany).

The identification and quantification of colonic aberrant crypt foci (ACF) were performed using methylene blue staining. Formalin-fixed colons were longitudinally incised along the median axis and then stained with a 2% methylene blue solution. The total number of aberrant crypts per rat and aberrant crypts per focus were evaluated under a light microscope.

### 4.6. Immunohistochemistry of Proliferation Cell Nuclear Antigen (PCNA)

Immunohistochemical staining to detect PCNA-positive cells was conducted following a method adapted from Punvittayagul et al., 2021 [65]. Liver and colon sections were initially deparaffinized, then immersed in 10 mM citrate buffer (pH 6.0) using autoclave. Afterward, the sections were incubated with monoclonal mouse anti-rat PCNA antibodies (BioLegend, San Diego, CA, USA), followed by biotinylated antibodies. Next, an Elite avidin-biotin complex kit was utilized along with the DAB reagent for visualization, with hematoxylin as a counterstain. The count of PCNA-positive cells was performed using a light microscope.

### 4.7. Terminal Deoxynucleotidyltransferase (TdT)–dUTP Nick End Labeling (TUNEL) Assay

Apoptotic cells within the liver and colon were identified using an ApopTag Peroxidase in situ kit, as previously described by Thumvijit et al., 2014 [66]. The procedures were executed following the instructions provided by the kit. The resultant color development was achieved through the utilization of DAB. The number of apoptotic cells was counted under a light microscope.

### 4.8. Determination of Antioxidant Enzyme Activities in Rat Liver

The frozen livers from non-carcinogens-treated rats (Groups 4–6) were used to investigate the effect of *P. emblica* fruits on antioxidant enzyme activities in the liver. The liver tissues were homogenized and centrifuged at 10,000 rpm for 20 min, and then ultracentrifuged at 100,000× *g* for 60 min to obtain a cytosolic supernatant fraction. The protein concentration was measured using the Lowry method. The activities of the antioxidant enzymes, including glutathione peroxidase (GPx), glutathione reductase (GR), and catalase (CAT), as well as the contents of total reduced glutathione (GSH) and oxidized glutathione (GSSG), were measured according to the method previously described [67].

### 4.9. Determination of Phytochemical Contents

The crude extract underwent phytochemical screening to assess the presence of alkaloids, saponins, steroids, terpenoids, polyphenols, flavonoids, tannins, and glycosides, using a previously described method [68]. Additionally, all solvent-partitioning fractions were analyzed for total phenolic, total flavonoid, and total condensed tannin contents using established methods [69]. The determination of total hydrolysable tannin was carried out using the potassium iodate method [70]. Quantitative analysis of specific phytochemicals, such as phenolic acids and certain flavonoids, was conducted using reverse-phase HPLC with an Agilent ZORBAX Eclipse Plus C18 column (4.6 × 250 mm, 5 μM) (Agilent Technologies, Santa Clara, CA, USA) at a flow rate of 1 mL/min. The phytochemical standard agents used included gallic acid, protocatechuic acid, 4-hydroxybenzoic acid, chlorogenic acid, vanillic acid, syringic acid, *p*-coumaric acid, ferulic acid, ellagic acid, and *trans*-cinnamic acid for phenolic acids, and catechin, epicatechin, rutin, quercetin, luteolin, apigenin, quercitrin, myricetin, naringenin, kaempferol, and isorhamnetin for flavonoids. To determine the concentration of phytochemical contents, HPLC analysis was employed by comparing the retention times (RTs) and peak areas of analytes to those of known standards [71,72]. The concentration of each component in the sample was quantified using a standard curve of each standard compound. Spike recovery experiments were conducted by adding known amounts of the standard to samples at different concentrations and then analyzing them using Agilent HPLC 1100 (Agilent Technologies, Santa Clara, CA, USA). The results were expressed as milligrams per gram extract.

### 4.10. Screening of Antioxidant Activity

2,2-Diphenyl-1-picrylhydrazyl (DPPH) radical scavenging assay, ABTS^•+^ radical scavenging assay, and ferric reducing antioxidant power (FRAP) assay were used to determine the degree of antioxidant activity according to the previously described method [73]. 

### 4.11. Cytotoxicity of Murine Macrophage Cells

The cytotoxicity of test samples on RAW 264.7 macrophages was evaluated using the 3-(4,5-dimethylthiazol-2-yl)-2,5-diphenyltetrazolium bromide (MTT) assay, as previously described [74]. RAW 264.7 cells were seeded at a density of 2 × 10^5^ cells/mL and treated with concentrations of the test compounds up to 1250 µg/mL for 24 h. Subsequently, the cells were incubated with MTT reagent (Sigma-Aldrich, St. Louis, MO, USA) at a final concentration of 0.5 mg/mL for two hours to determine cell viability. The absorbance of the solubilized formazan was then measured at 540 nm and 630 nm. The results were expressed as IC_20_ values, with doses below the IC20 values considered non-cytotoxic.

### 4.12. Antioxidant Activity in Murine Macrophage Cells

RAW 264.7 cells at 2 × 10^5^ cells/mL were treated with various concentrations of test samples for 24 h and then stimulated with LPS for another 24 h. Afterward, cells were incubated with DCFHDA for 30 min. The fluorescence signal was then measured by Agilent BioTek Synergy H4 Hybrid Microplate Reader ( Winooski, VT, USA) at an excitation wavelength of 485 nm and an emission wavelength of 530 nm [75].

### 4.13. Anti-Inflammatory Activity in Murine Macrophage Cells

The anti-inflammatory activity of each fraction was determined following a previously described procedure [74]. Briefly, RAW 264.7 cells at 2 × 10^5^ cells/mL were pretreated with various concentrations of the test samples for 24 h, followed by treatment with LPS for another 24 h. The culture supernatant was collected to determine the nitrite concentration by adding Griess reagent (Sigma-Aldrich, St. Louis, MO, USA), with absorbance subsequently measured at 550 nm.

### 4.14. Mutagenic and Antimutagenic Properties

The experiment utilized *Salmonella typhimurium* strains TA 98 and TA100, kindly provided by Dr. Kei-Ichi Sugiyama of the National Institute of Health, Tokyo, Japan. The conditions with and without metabolic activation achieved from rat hepatic post-mitochondrial fraction (S9) (Sigma-Aldrich, St. Louis, MO, USA) were employed to detect indirect and direct mutagenic and antimutagenic properties, respectively [69].

For mutagenicity evaluation, a test sample was combined with overnight culture bacteria and sodium phosphate buffer (−S9) or S9 mixture (+S9). After incubation at 37 °C for 20 min, top agar was added, and the mixture was further incubated at 37 °C for another 48 h. Positive controls, including 2AA and AF-2, were used in the presence and absence of metabolic activation, respectively. The number of revertant colonies was counted and expressed as a mutagenic index (MI).

To investigate antimutagenicity, non-cytotoxic doses of each fraction were mixed with overnight culture bacteria and a particular standard mutagen. Under the S9 condition, AFB_1_ (50 ng/pl) and MelQ (25 ng/pl) were employed as standard mutagens for the TA98 and TA100 strains, respectively. In the absence of the S9 condition, AF-2 (0.1 µg/pl) and NaN_3_ (1 µg/pl) served as standard mutagens for the TA98 and TA100 strains, respectively. The number of revertant colonies was then counted and the results were expressed as percentages of inhibition.

### 4.15. Statistical Analysis

The results were expressed as mean ± SD values. Statistical comparisons between groups were analyzed using one-way ANOVA followed by Bonferroni post hoc analysis using GraphPad Prism 9.0 software (GraphPad Software, Boston, MA, USA). Statistical significance is denoted as * *p* ≤ 0.05, ** *p* ≤ 0.01, *** *p* ≤ 0.001, **** *p* ≤ 0.0001.

## 5. Conclusions

In conclusion, the ethanolic extract of *P. emblica* fruits serves as a potent chemopreventive agent in a dual-carcinogen model, effectively inhibiting the development of preneoplastic lesions in both the liver and colon. These effects are mediated through the modulation of antioxidant defenses, xenobiotic metabolizing enzymes, and inflammatory responses. Moreover, the study emphasizes the importance of potentially possible active compounds in the DCM fraction. Finally, these findings support the use of *P. emblica* fruit and its potential active compounds as supplements for cancer chemoprevention.

While these results are promising concerning the effect of *P. emblica* fruit in a strong model of co-carcinogen exposure, there are several limitations. Further elucidation of the mechanisms by which the extract influences cellular pathways is needed in both animal models and human populations to more accurately replicate human responses. Moreover, in vitro results often do not align with the findings of in vivo studies [76,77]. Therefore, confirming the active compounds in *P. emblica* fruit extract through studies in animal models remains challenging.

## Figures and Tables

**Table 1:** Spectrophotometric and HPLC fingerprint analysis of phytochemical constituents of crude ethanolic extract of *P. emblica* fruits and its fractions.

	Crude	HEX	DCM	EAC	BA	Residue
**Spectrophotometric analysis**						
Total phenolic (mg GAE/g)	530.49 ± 20.36	181.53 ± 9.86	337.58 ± 26.76	853.04 ± 47.73	517.59 ± 22.46	301.92 ± 37.98
Total flavonoid (mg CE/g)	19.58 ± 4.05	22.53 ± 4.40	30.82 ± 3.57	36.34 ± 3.32	21.90 ± 1.15	7.73 ± 5.55
Total condensed tannin (mg CE/g)	5.19 ± 0.30	0.73 ± 0.08	0.89 ± 0.03	1.52 ± 0.62	1.01 ± 1.30	ND
Total hydrolysable tannin (mg MGE/g)	47.28 ± 4.96	33.64 ± 1.48	169.20 ± 2.49	329.42 ± 3.28	36.19 ± 2.09	10.90 ± 1.43
**HPLC analysis**						
Gallic acid (mg/g)	80.25 ± 5.09	31.26 ± 0.01	36.22 ± 2.22	281.03 ± 1.37	27.75 ± 0.08	ND
Syringic acid (mg/g)	1.19 ± 0.01	ND	0.86 ± 0.01	2.60 ± 0.09	ND	ND
Ellagic acid (mg/g)	3.54 ± 0.05	11. 82 ± 0.22	14.96 ± 0.74	6.98 ± 0.15	5.69 ± 0.21	2.86 ± 0.25
*trans*-cinnamic acid (mg/g)	ND	13.76 ± 0.01	56.12 ± 0.13	ND	ND	ND
Catechin (mg/g)	1.56 ± 0.01	ND	ND	ND	ND	ND
Epicatechin (mg/g)	76.12 ± 0.01	14.65 ± 0.04	28.89 ± 0.05	35.71 ± 0.02	47.20 ± 0.20	ND
Rutin (mg/g)	0.32 ± 0.02	0.03 ± 0.01	0.15 ± 0.05	0.15 ± 0.01	11.82 ± 0.04	ND

The results are presented as mean ± standard deviation (SD) values. ND: Not Detectable, GAE: Gallic acid equivalents, CE: Catechin equivalents, MGE: Methyl gallate equivalents.

**Table 2:** Antioxidant activity of crude ethanolic extract of *P. emblica* fruits and its fractions.

Sample	DPPH (IC_50_) (μg/mL)	ABTS (IC_50_) (μg/mL)	FRAP (μM TE/g Extract)
Crude	51.92 ± 15.76	254.71 ± 103.39	4199.24 ± 317.86
HEX	898.40 ± 133.55	2555.58 ± 377.31	171.02 ± 30.31
DCM	126.80 ± 75.64	2417.85 ± 109.93	2687.16 ± 177.89
ECA	30.53 ± 6.61	100.90 ± 30.79	5423.04 ± 460.77
BA	54.64 ± 3.72	210.62 ± 13.51	3244.69 ± 256.60
Residue	98.21 ± 26.11	247.61 ± 40.44	1678.49 ± 52.73

The values were expressed as mean ± SD obtained from three experiments. TE: Trolox equivalents.

**Table 3:** Mutagenicity of crude ethanolic extract of *P. emblica* fruits and its fractions using *Salmonella* mutation assay.

Sample	Dose (mg/plate)	Average of His+ Revertant Colonies per Plate (MI)			
		TA98		TA100	
		+S9	−S9	+S9	−S9
DMSO		34.0 ± 1.5	34.8 ± 0.4	121.0 ± 6.4	116.8 ± 4.6
2-AA	0.0005	1553.1 ± 84.8 (45.7)	−	909.9 ± 13.9 (7.5)	−
AF-2	0.0001	−	413.0 ± 36.7 (11.9)	−	−
AF-2	0.00001	−	−	−	882.3 ± 49.3 (7.6)
Crude	0.2	36.0 ± 1.7 (1.1)	26.9 ± 0.7 (0.8)	130.7 ± 5.0 (1.1)	106.8 ± 1.9 (0.92)
	1	35.4 ± 2.6 (1.0)	27.8 ± 1.5 (0.8)	112.8 ± 11.0 (0.9)	112.3 ± 2.9 (1.0)
	5	29.9 ± 0.4 (0.9)	25.9 ± 1.2 (0.7)	107.8 ± 4.9 (0.9)	99.7 ± 7.3 (0.9)
HEX	0.2	33.1 ± 2.4 (1.0)	27.2 ± 0.8 (0.8)	121.9 ± 1.4 (1.0)	100.7 ± 6.7 (0.9)
	1	30.9 ± 1.6 (0.9)	27.4 ± 1.0 (0.8)	88.3 ± 8.2 (0.7)	80.2 ± 4.0 (0.7)
	5	33.3 ± 0.5 (1.0)	26.2 ± 0.6 (0.8)	86.7 ± 4.8 (0.7)	76.2 ± 1.09 (0.7)
DCM	0.2	39.1 ± 1.4 (1.2)	28.1 ± 0.5 (0.8)	112.2 ± 4.2 (0.9)	113.6 ± 7.6 (1.0)
	1	32.9 ± 1.4 (1.0)	27.4 ± 1.0 (0.8)	109.7 ± 7.5 (0.9)	112.6 ± 0.9 (1.0)
	5	33.7 ± 2.2 (1.0)	26.2 ± 0.6 (0.8)	107.1 ± 4.3 (0.9)	110.8 ± 3.2 (1.0)
EAC	0.2	38.2 ± 1.2 (1.1)	27.1 ± 1.1 (0.8)	116.1 ± 4.8 (1.0)	115.8 ± 1.2 (1.0)
	1	50.4 ± 3.1 (1.5)	28.0 ± 0.8 (0.8)	106.2 ± 3.5 (0.9)	94.3 ± 1.4 (0.8)
	5	46.2 ± 2.1 (1.4)	30.4 ± 0.1 (0.9)	96.2 ± 1.5 (0.8)	97.4 ± 4.0 (0.8)
BA	0.2	34.7 ± 1.2 (1.0)	24.8 ± 1.9 (0.7)	105.7 ± 8.2 (0.9)	108.3 ± 4.9 (0.9)
	1	31.9 ± 2.0 (0.9)	27.2 ± 0.6 (0.8)	107.7 ± 6.9 (0.9)	112.8 ± 10.4 (1.0)
	5	27.0 ± 1.0 (0.8)	28.2 ± 0.8 (0.8)	102.0 ± 4.9 (0.8)	118.0 ± 4.3 (1.0)
Residue	0.2	34.8 ± 1.4 (1.0)	24.0 ± 1.5 (0.7)	108.2 ± 3.5 (0.9)	114.8 ± 2.1 (1.0)
	1	34.4 ± 1.3 (1.0)	26.7 ± 1.5 (0.8)	104.8 ± 5.3 (0.9)	105.2 ± 2.0 (0.9)
	5	26.2 ± 1.2 (0.8)	26.6 ± 0.6 (0.8)	126.5 ± 21.9 (1.0)	125.9 ± 5.1 (1.0)

Values are expressed as mean ± SD. 2AA: 2-aminoanthracene. AF-2: 2-(2-furyl)-3-(5-nitro-2-furyl)acrylamide.

**Table 4:** Antimutagenicity against direct-, and indirect-acting mutagens of crude ethanolic extract of *P. emblica* fruits and its fractions using *Salmonella* mutation assay.

Sample	Dose (mg/plate)	Average of His^+^ Revertant Colonies per Plate (%Inhibition)			
		TA98		TA100	
		+S9	−S9	+S9	−S9
DMSO		26.3 ± 4.8 *	24.6 ± 0.6 *	125.9 ± 1.3 *	107.2 ± 6.0 *
AFB_1_	0.00005	926.7 ± 70.6	−	−	−
AF-2	0.0001	−	324.6 ± 2.1	−	−
MeIQ	0.000025	−	−	1482.6 ± 103.4	−
NaN_3_	0.001	−	−	−	498.4 ± 74.2
Crude	0.04	744.2 ± 21.4 (19.4)	256.0 ± 4.7 (22.8)	1199.3 ± 105.6 (21.1)	419.8 ± 40.8 (18.4)
	0.2	613.6 ± 11.2 (34.0) *	242.6 ± 8.5 (27.4)	1055.7 ± 51.2 (31.0) *	381.8 ± 50.4 (29.5)
HEX	0.04	541.7 ± 44.3 (42.8) *	250.2 ± 2.1 (24.8)	916.2 ± 75.1 (41.9) *	450.7 ± 36.5 (8.3)
	0.2	108.1 ± 2.4 (90.9) *^,#^	237.1 ± 15.8 (29.4)	509.0 ± 89.8 (72.2) *^,#^	423.6 ± 41.7 (16.3)
DCM	0.04	435.3 ± 3.3 (54.0) *^,#^	240.4 ± 30.4 (28.1)	772.7 ± 51.0 (52.3) *^,#^	448.8 ± 42.0 (7.8)
	0.2	134.9 ± 5.5 (87.9) *^,#^	250.3 ± 7.7 (24.7)	434.7 ± 8.2 (77.0) *^,#^	406.2 ± 34.6 (20.6)
EAC	0.04	877.6 ± 40.6 (4.8)	258.9 ± 14.3 (21.9)	1269.1 ± 105.7 (15.9)	478.9 ± 52.4 (1.8)
	0.2	208.1 ± 40.8 (79.0) *^,#^	244.0 ± 16.0 (26.8)	583.3 ± 49.8 (66.4) *^,#^	376.2 ± 32.1 (28.6)
BA	0.04	772.0 ± 21.0 (16.5)	236.1 ± 5.5 (29.5)	1260.3 ± 101.6 (16.5)	458.7 ± 41.7 (7.7)
	0.2	321.3 ± 49.7 (66.2) *^,#^	235.4 ± 8.8 (29.7)	940.0 ± 29.6 (39.5) *	424.9 ± 35.3 (16.5)
Residue	0.04	593.1 ± 35.8 (35.7) *	236.1 ± 14.9 (29.5)	1282.9 ± 76.0 (14.6)	463.1 ± 61.0 (8.0)
	0.2	543.3 ± 55.1 (40.8) *	242.2 ± 15.6 (27.4)	888.4 ± 54.2 (43.3) *	464.2 ± 54.3 (7.5)

Values are expressed as mean ± SD. * Significant difference from mutagen control (*p* ≤ 0.05). ^#^ Significant difference from other significant fractions at the same dose and conditions (*p* ≤ 0.05). AFB_1_: aflatoxins B_1_. AF-2: 2-(2-furyl)-3-(5-nitro-2-furyl)acrylamide. MelQ: 2-amino-3,4-dimethylimidazo[4,5-f]quinoline. NaN_3_: Sodium azide.

**Table 5:** Antimutagenicity against direct-, and indirect-acting mutagens of potential leading bioactive compounds of DCM fraction using *Salmonella* mutation assay.

Sample	Dose (mg/plate)	Average of His^+^ Revertant Colonies per Plate (%Inhibition)	
		TA98 (+S9)	TA100 (+S9)
DMSO		26.7 ± 1.0 *	116.3 ± 2.5 *
AFB_1_	0.00005	965.0 ± 37.9	−
MeIQ	0.000025	−	1024.0 ± 56.5
Vanillic acid	1	417.0 ± 15.1 (58.4) *	466.0 ± 19.5 (61.1) *
DCM	0.2	79.8 ± 5.2 (94.4) *	207.7 ± 23.1 (90.0) *
tCA	0.01	891.0 ± 39.7 (7.9)	998.7 ± 54.3 (2.8)
	0.2	888.3 ± 7.8 (7.9)	842.7 ± 11.3 (19.5)
	1	608.7 ± 37.5 (37.9) *	509.3 ± 35.1 (56.5) *
EA	0.003	890.7 ± 33.2 (10.1)	995.0 ± 47.9 (3.1)
	0.2	622.7 ± 32.7 (36.5) *	745.0 ± 32.6 (30.1) *
	1	403.3 ± 18.7 (59.6) *	356.0 ± 11.3 (73.4) *

Values are expressed as mean ± SD. * Significant difference from mutagen control (*p* ≤ 0.05). Vanillic acid at 1 mg/plate served as positive control. AFB_1_: aflatoxins B_1_. AF-2: 2-(2-furyl)-3-(5-nitro-2-furyl)acrylamide. MelQ: 2-amino-3,4-dimethylimidazo[4,5-f]quinoline. NaN_3_: Sodium azide. tCA: *trans*-cinnamic acid. EA: Ellagic acid.

## Data Availability

The authors declare that the majority of the data supporting their findings are available in the information files of the paper. Any inquiries regarding the data can be directed to the corresponding author.

## Supplementary Materials

The following supporting information can be downloaded at: https://www.mdpi.com/article/10.3390/ph17070818/s1, Table S1: Qualitative phytochemical screening of the phytochemical constituents of crude ethanolic extract of *P. emblica*; Table S2: IC_20_ of crude ethanolic extract of *P. emblica*. and its fractions in Raw 264.7 cell lines; Figure S1: Chromatograms of the phenolic compounds in *P. emblica* fruit extract and its fractions; Figures S2 & S3: Chromatograms of the flavonoid compounds in *P. emblica* fruit extract and its fractions.
