# Supplementary material for: Chemopreventive Potential of Phyllanthus emblica Fruit Extract against Colon and Liver Cancer Using a Dual-Organ Rat Carcinogenesis Model
Source: Pharmaceuticals (Basel). 2024 Jun 21;17(7):818. doi: 10.3390/ph17070818 (PMC11280025; doi:10.3390/ph17070818)
Supplement: Supplementary file 1 [file pharmaceuticals-17-00818-s001.zip › pharmaceuticals-3059120-supplementary.pdf]

pharmaceuticals

Chemopreventive Potential of *Phyllanthus emblica* Fruit Extract against Colon and Liver Cancer Using a Dual-Organ Rat Carcinogenesis Model

Chonikarn Singai [1], Pornsiri Pitchakarn [1], Sirinya Taya [2], Warunyoo Phannasorn [1], Rawiwan Wongpoomchai [1] and Ariyaphong Wongnoppavich [1,*]

[1] Department of Biochemistry, Faculty of Medicine, Chiang Mai University, Chiang Mai 50200, Thailand; chonikarn.s@gmail.com (C.S.); pornsiri.p@cmu.ac.th (P.P.); p.warunyoo@gmail.com (W.P.); rawiwan.wong@cmu.ac.th (R.W.)
[2] Functional Food Research Unit, Multidisciplinary Research Institute, Chiang Mai University, Chiang Mai 50200, Thailand; sirinya.t@cmu.ac.th
* Correspondence: ariyaphong.w@cmu.ac.th

Supplementary Table S1. Qualitative phytochemical screening of the phytochemical constituents of crude ethanolic extract of *P. emblica.*

Groups of Phytochemicals	Test/ Reagent	Results
Alkaloids	Wagner's test	-
Saponins	Foam test	+
Steroids/sterols	Liebermann-Burchard test	-
Terpenoids	Salkowski's test	+
Polyphenols	Ferric Chloride test	+++
Flavonoids	Ammonia solution	++
Tannins	Ferric chloride test	+++
Cardiac glycoside	Keller-Killiani test	+

+++: strong intensity reaction, ++: medium intensity reaction, +: weak intensity reaction,

-: non-detected

Supplementary Table S2. IC_{20} of crude ethanolic extract of *P. emblica*. and its fractions in Raw 264.7 cell lines

Sample	IC_{20} (μg/mL)
Crude	377.27
HEX	267.15
DCM	110.66
EAC	271.65
BA	696.11
Residue	≥1250

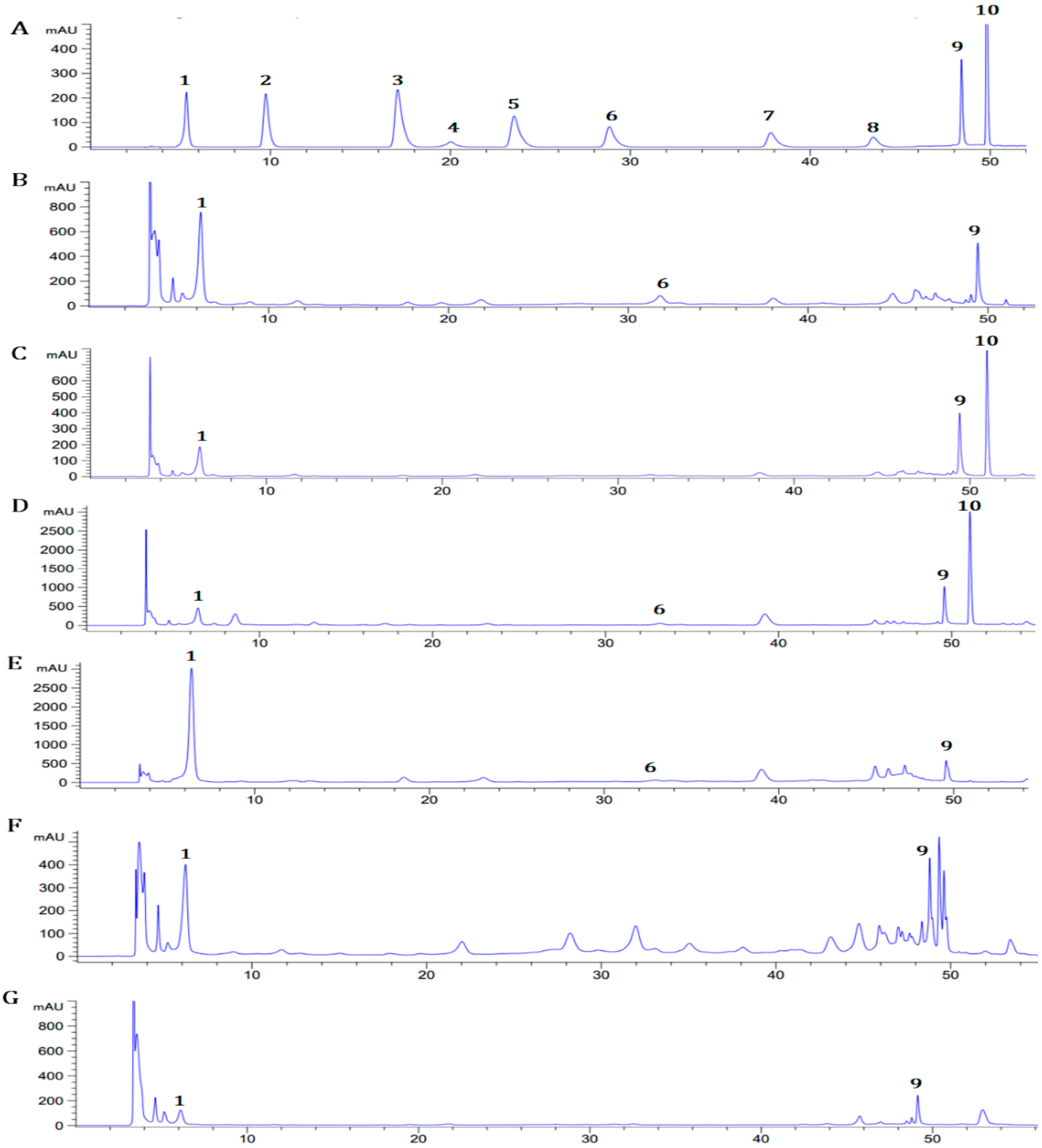

Supplementary Figure S1. Chromatograms of the phenolic compounds in *P. emblica* fruit extract and its fractions. The detection of phenolic compounds: (**1**) gallic acid, (**2**) protocatechuic acid, (**3**) 4-hydroxybenzoic acid, (**4**) chlorogenic acid, (**5**) vanillic acid, (**6**) syringic acid, (**7**) *p*-coumaric acid, (**8**) ferulic acid, (**9**) ellagic acid, and (**10**) *trans*-cinnamic acid in the samples: (**A**) mixture of standard phenolic compounds, (**B**) crude extract, (**C**) HEX, (**D**) DCM, (**E**) ECA, (**F**) BA, and (**G**) Residue. Absolute methanol and 3% acetic acid in water were used as mobile phases.

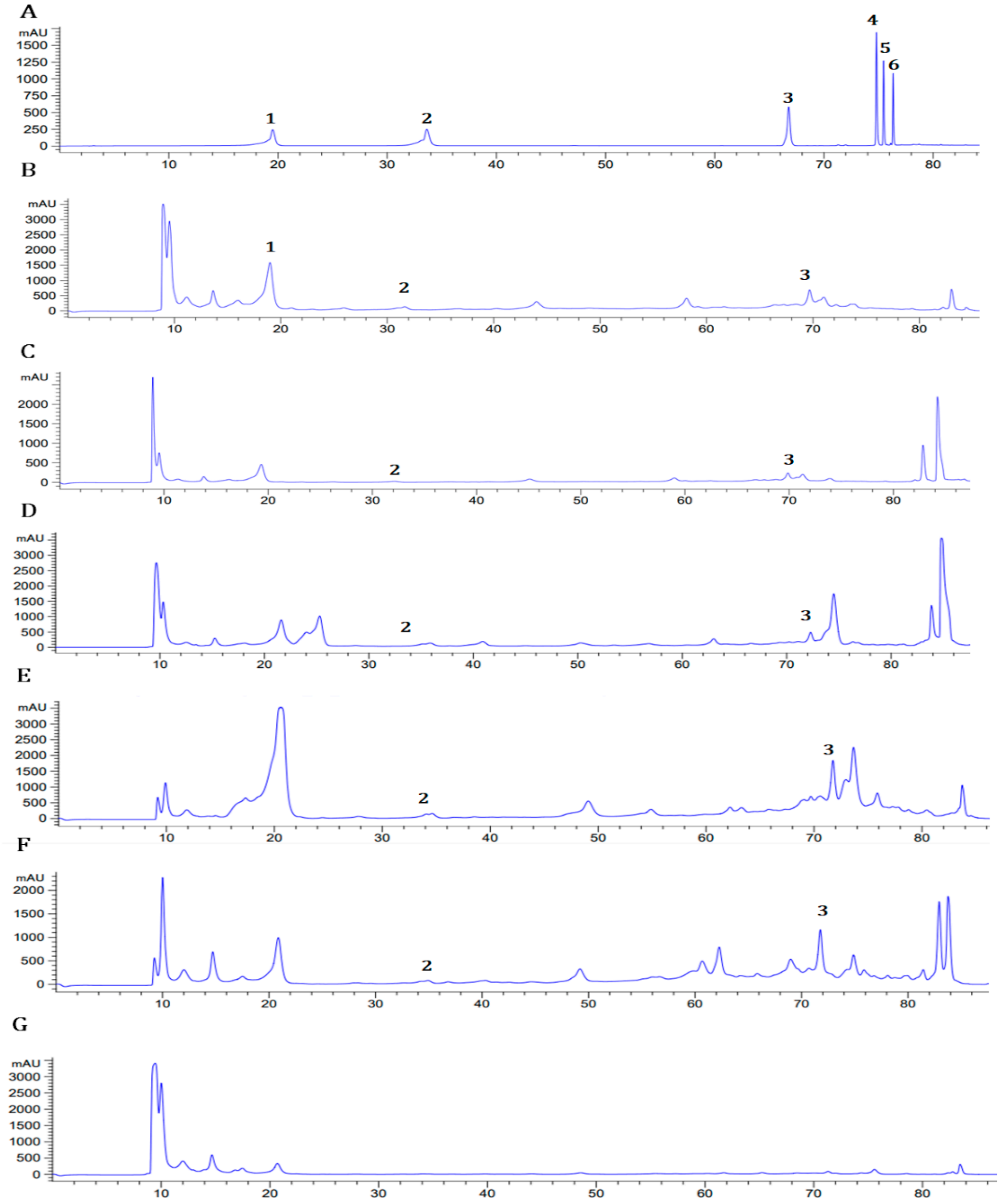

Supplementary Figure S2. Chromatograms of the flavonoid compounds in *P. emblica* fruit extract and its fractions. The detection of flavonoid compounds: (**1**) catechin, (**2**) epicatechin, (**3**) rutin, (**4**) quercetin, (**5**) luteolin, and (**6**) apigenin in the samples: (**A**) mixture of standard flavonoid compounds, (**B**) crude extract, (**C**) HEX, (**D**) DCM, (**E**) ECA, (**F**) BA, and (**G**) Residue. 1% acetic acid in water and 1% acetic acid in MeOH were used as mobile phases.

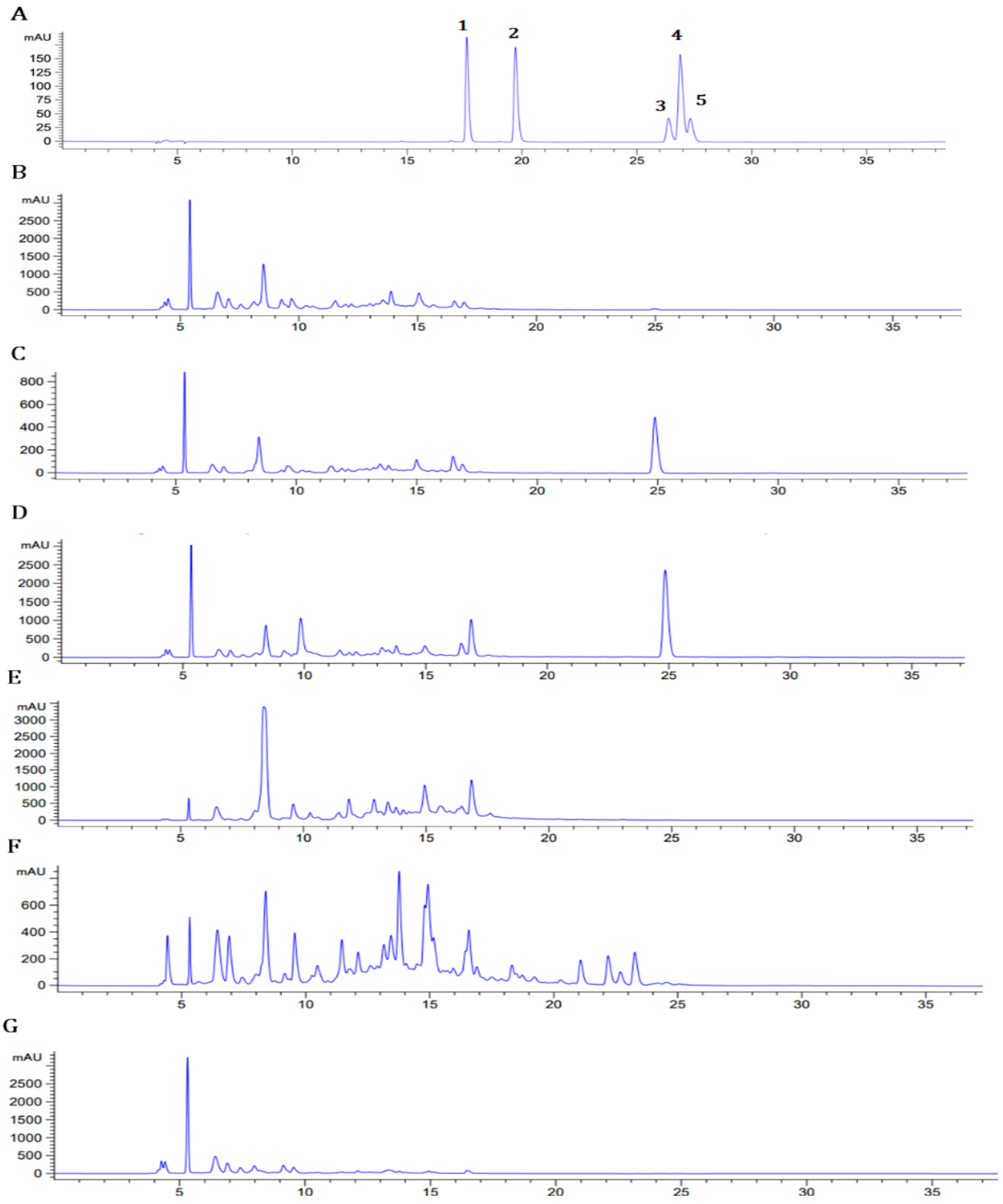

Supplementary Figure S3. Chromatograms of the flavonoid compounds in *P. emblica* fruit extract and its fractions. The detection of flavonoid compounds: (**1**) quercitrin, (**2**) myricetin, (**3**) naringenin, (**4**) kaempferol, and (**5**) isorhamnetin in the samples: (**A**) mixture of standard flavonoid compounds, (**B**) crude extract, (**C**) HEX, (**D**) DCM, (**E**) ECA, (**F**) BA, and (**G**) Residue. 0.2% formic acid in water and acetonitrile were used as mobile phases.
